# 707. Hospital-Onset *Clostridioides difficile* Infection Rates During COVID-19 Pandemic in the ICU Patients

**DOI:** 10.1093/ofid/ofab466.904

**Published:** 2021-12-04

**Authors:** Evdokia Gavrielatou, prodromos Temperikidis, Michalis Tsimaras, eleni magira

**Affiliations:** 1 National Kapodistrian University of Athens, Athens, Attiki, Greece; 2 University of Athens Medical School, athens 15235, Attiki, Greece

## Abstract

**Background:**

Due to COVID-19 gastrointestinal microbiome alterations, COVID-19 can be complicated by *Clostridioides difficile* infection (CDI). This retrospective cohort study aimed to evaluate the prevalence of *Clostridium difficile* infection in patients with COVID-19pneumonia

**Methods:**

A retrospective cohort study was conducted on PCR Covid-19 positive patients admitted in the ICU from September,2020 to 30^th^ April 2021. All patients in the cohort study were on mechanical ventilation, or at some point during their ICU admission required mechanical ventilation. Hospital-onset (HO-CDI), defined as a positive *C*. *difficile* test over 3 days after admission.

**Results:**

Overall, during the study period, a total of 240 PCR Covid-19 patients were admitted to the ICU; of these, 11 (4.5%) were COVID-19 CDI positive. Nine were males (81%). The mean hospital stay for these COVID-19 patients was 12 days (range 1–59 days). HO-CDI median day of identification was 12 days. All patients received ≥2 antibiotics and dexamethasone at admission. Compared to historical controls, COVID-19 patients did not have a higher overall CDI positive rate. However, mortality among COVID-19 HO-CDI patients was increased 7/11 (63%).

**Conclusion:**

Whether COVID-19 itself increases an individual’s risk for CDI remains unclear. Multiple contributing factors drive CDI incidence, severity, and recurrence. Although protective measures such as gowns and gloves during COVID-19 increased, CDI cases in the hospital setting should continue to emphasize the importance of antimicrobial stewardship.

**Disclosures:**

**All Authors**: No reported disclosures

